# Spontaneous coronary artery dissection during cisplatin and capecitabine therapy

**DOI:** 10.1016/j.amsu.2019.07.018

**Published:** 2019-07-11

**Authors:** Pavel Somov, Dmitriy Marchak, Arkhip Matusov, Alexander Viller, Yuri Shevchenko, Archili Miminoshvili

**Affiliations:** a Pirogov's National Medical Surgical Center, Moscow, Russian Federation; b Institute of Urgent and Recovery Surgery of V.K.Gusak, Ukraine

**Keywords:** Spontaneous coronary artery dissection, Myocardial infarction, Capecitabine, Cisplatin

## Abstract

**Introduction:**

Spontaneous coronary artery dissection is a rare cardiovascular disease that can cause acute myocardial infarction and sudden cardiac death. The mechanism of this pathology and the optimal treatment are not fully understood.

**Presentation of case:**

An acute myocardial infarction developed while a fifty five years old woman with a rectal adenocarcinoma was receiving a cisplatin and capecitabine therapy. Coronary angiography demonstrated a multivessel occlusion of coronary arteries.

**Discussion:**

The authors discuss several factors that may lead to the spontaneous coronary artery dissection including chemotherapy-induced vasospasm. Chemotherapy based on the cisplatin and capecitabine intake can cause a cardiotoxic effect.

**Conclusion:**

Thus, spontaneous coronary artery dissection is a disease with an extremely complex etiololy, which does not have a special treatment guide. Management should be considered individually for each case.

## Highlights

• SCAD is a rare cardiovascular disease that can cause acute myocardial infarction and sudden cardiac death.
• Cisplatin and capecitabine therapy is a risk factor for SCAD.
• One of the most important side effects of chemotherapy is cardiotoxicity.
• Management should be considered individually for each case of SCAD.

## 1 Introduction

Spontaneous coronary artery dissection (SCAD) is a very rare cardiovascular disease that due to the stratification of the coronary artery wall with a false lumen formation can lead to acute myocardial infarction (AMI) or less often to a rhythm disturbance and sudden heart death.

The first case of SCAD was described by N. Pretty in 1931. A fouty years old woman felt chest pain and in her left hand while preparing breakfast, after that she died while being hospitalised by an ambulance. At the autopsy, typical changes for cardiovascular pathology were absent.

There were minimal atherosclerotic changes in the coronary arteries. Fragments of a fresh blood clots and a second false lumen in the left anterior descending artery (LAD), were found [1].

The main mechanisms for the development of SCAD are intramural hematoma or damage of the intima, and not a ruptured atherosclerotic plaque. At present, the etiology of SCAD is not completely clear and, as a rule, in most cases it is explained by the predisposition to SCAD and a variety of trigger factors [2,3].

The following concomitant conditions and provoking factors that may be the cause of SCAD should be highlighted: pregnancy and the postpartum period, hormone therapy, fibromuscular dysplasia, autoimmune and inflammatory diseases, Takotsubo cardiomyopathy, depression, coronary tortuosity, genetic predisposition. The most frequent trigger mechanisms for the development of SCAD are excessive physical exercises and emotional stress.

As a rule, SCAD occurs in the middle aged women, regardless of the presence of cardiovascular risk factors. In rare cases, SCAD occurs during or shortly after pregnancy. When making a diagnosis of SCAD, it is advisable to exclude possible concomitant diseases. The case report has been prepared in line with the SCARE criteria [4].

## 2 Presentation of Case

In 2016, a fifty five years old woman with complaints of pain in the hip joints and the associated restriction of physical activity was hospitalised for further examination and total hip arthroplasty.

From the anamnesis, it is known that in April 2014, the patient underwent a course of chemotherapy for rectal adenocarcinoma with cisplatin and capecitabine. In August 2014, the patient suddenly felt a chest pain for the first time in her life.

Based on the clinical presentation and ECG data of acute myocardial infarction with ST segment elevation (ST segment elevation in I, II, aVL and V1–V4 up to 3–4 mm), the patient received thrombolytic therapy (metalyse 10000 units) after 60 minutes from the pain onset.

**Fig. 1:**
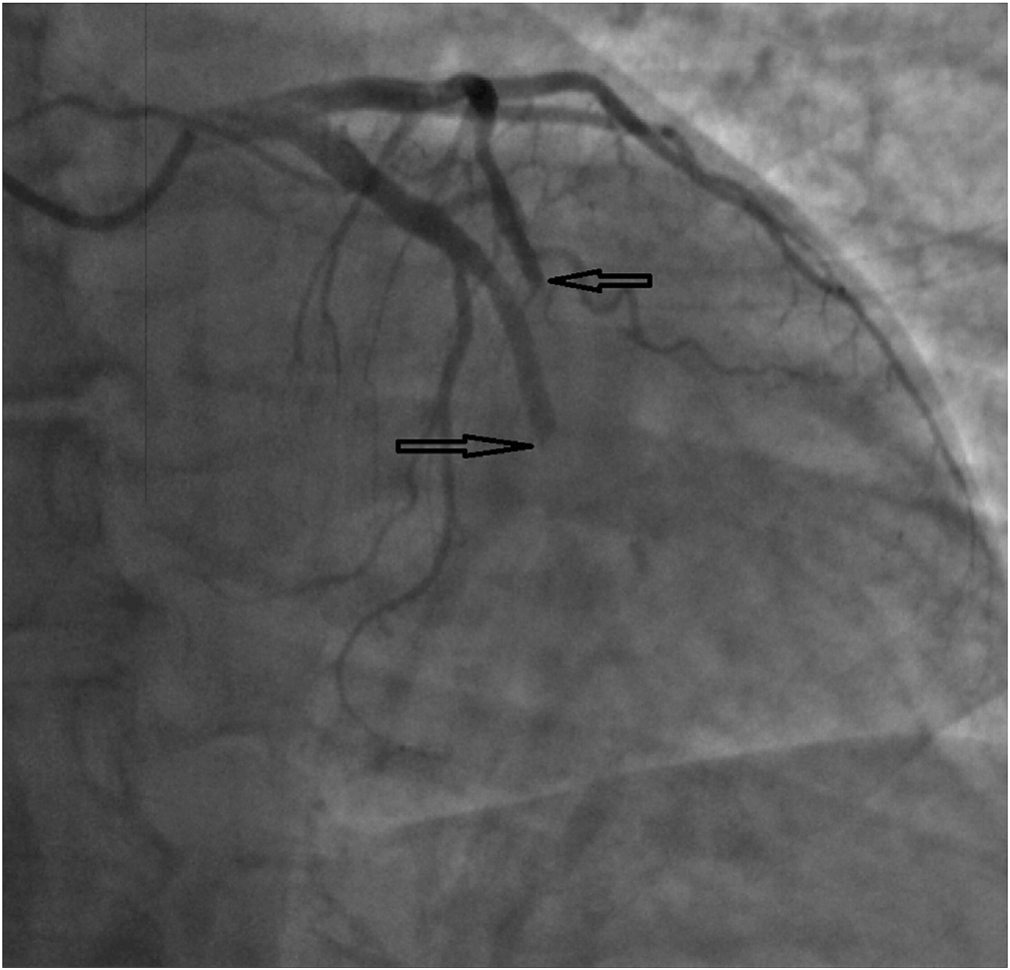
LAD and CxA occlusion.

**Fig. 2:**
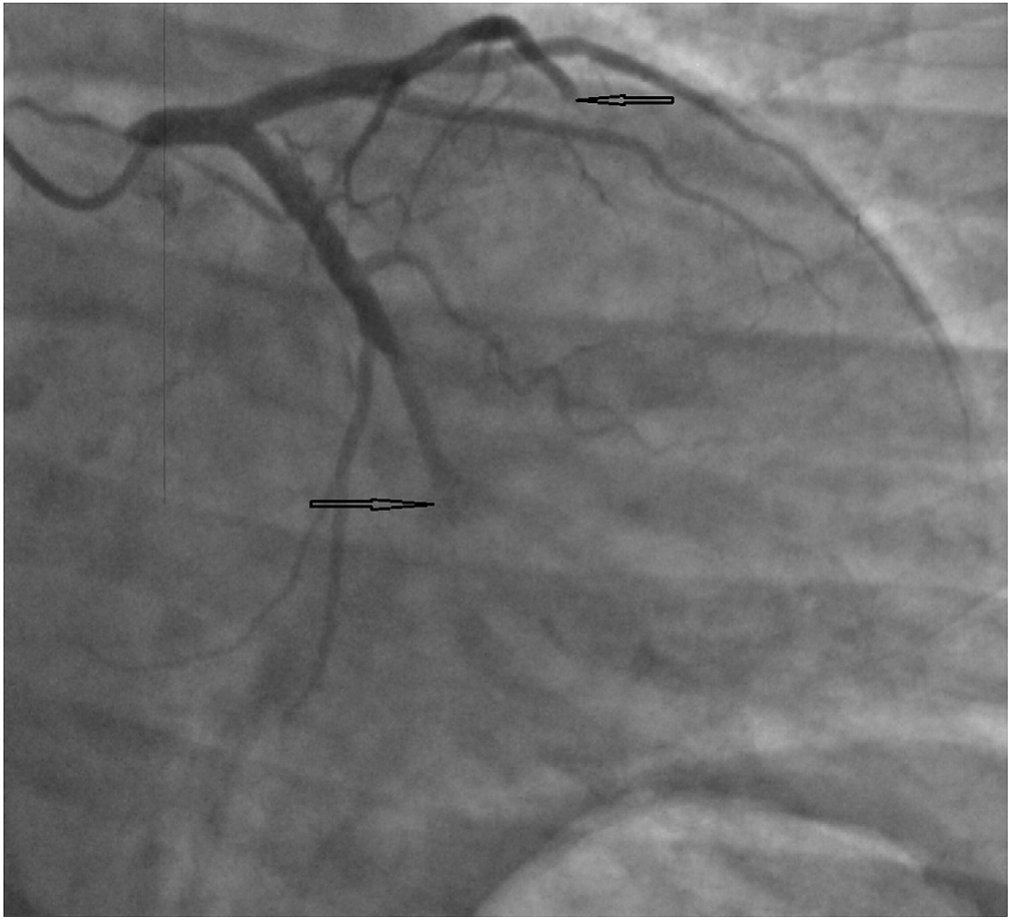
LAD and CxA occlusion.

After being hospitalised, urgent echocardiography (EchoCG) and coronary angiography (CAG) were performed. According to the CAG data, the presence of a multivessel coronary artery occlusion (LAD and circumflex artery) was revealed (Fig. 1, Fig. 2). It was decided to refrain from performing emergency percutaneous coronary intervention (PCI).

The results of the control echocardiogram before discharge the patient from the hospital: ejection fraction (EF) - 41%; decreased contractility of the left ventricle (LV) - akinesia of the LV apex, hypokinesis with areas of akinesis of the anterior septum, lower septum, basal segments of the anterior, anterolateral wall of the LV; thrombus in the apex 37 × 23 mm; LV apex aneurysm.

After applying the appropriate therapy, the patient condition became stable and she was discharged from the hospital in a satisfactory condition. For the next two years, the patient did not notice angina pectoris, she endured physical exertion satisfactorily, being limited by concomitant bilateral deforming osteoarthritis of the hip joints. According to the recommendation of oncologists, the patient completed the first chemotherapy course with cisplatin and capecitabine, and then stopped the therapy.

Given the history, the patient was performed radionuclide myocardial perfusion scintigraphy. The scintigraphic signs of cicatricial changes in the apex with an extension to apical segments of the front wall, the anterior interventricular septum (IVS), and in the posteriolateral walls (basal and middle segments), signs of aneurysm in the apex of the LV (aneurysm area-up to 10%); total area of cicatricial lesions, including aneurysm, about 35%, were revealed.

**Fig. 3:**
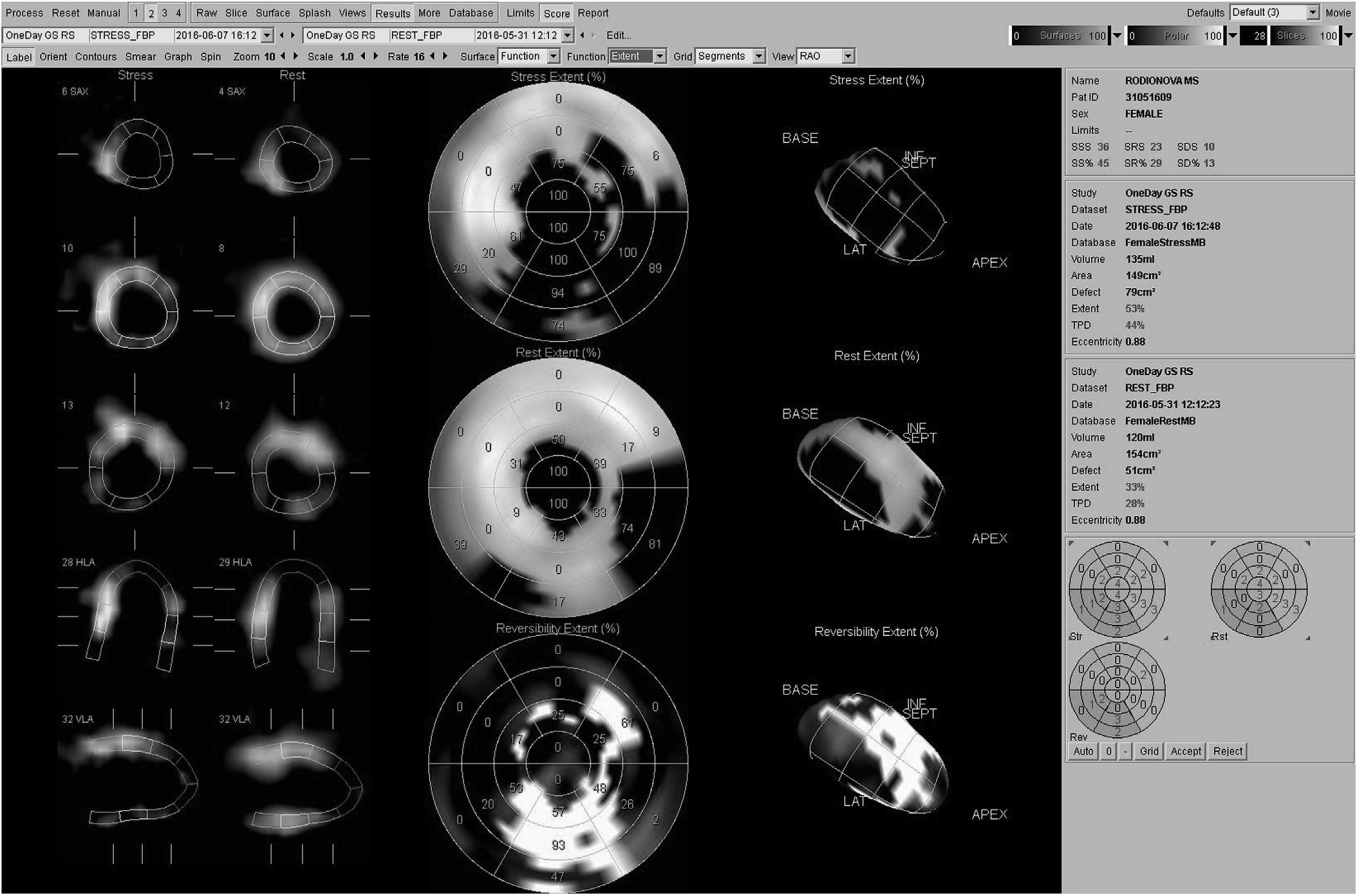
Radionuclide myocardial perfusion scintigraphy results.

A viable myocardium in the cicatricial zone was visualized in all the indicated segments, with the exception of the LV apex. The stress test revealed signs of stress-induced hipoperfusion in the cicatricial and adjacent zones: anterolateral wall (all levels), anterior wall, anterior interventricular septum (apical segments), anteriolateral wall (apical segments), as well as in the posterior wall (all levels); the ischemia zone was about 30%, the contractile ability of the LV myocardium was reduced (EF - 38%), signs of a decrease in systolic thickening in the cicatricial zone, with lower indices in the LV apex, was also determined (Fig. 3).

**Fig. 4:**
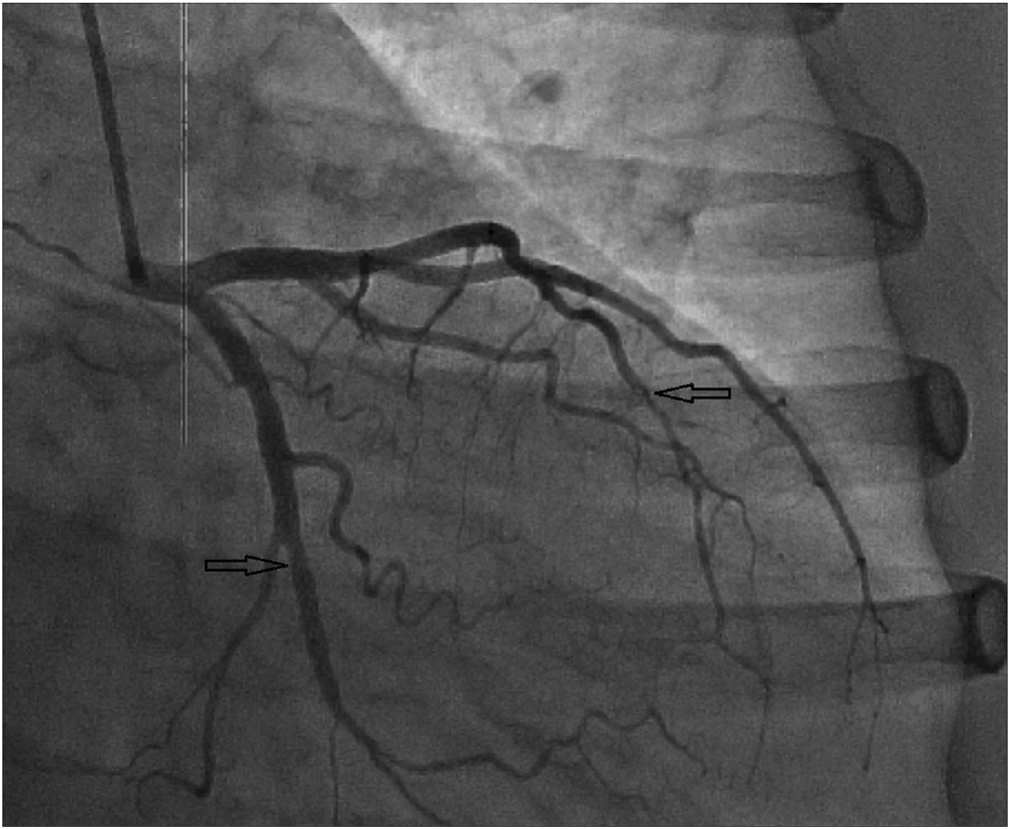
LAD and CA dissection.

**Fig. 5:**
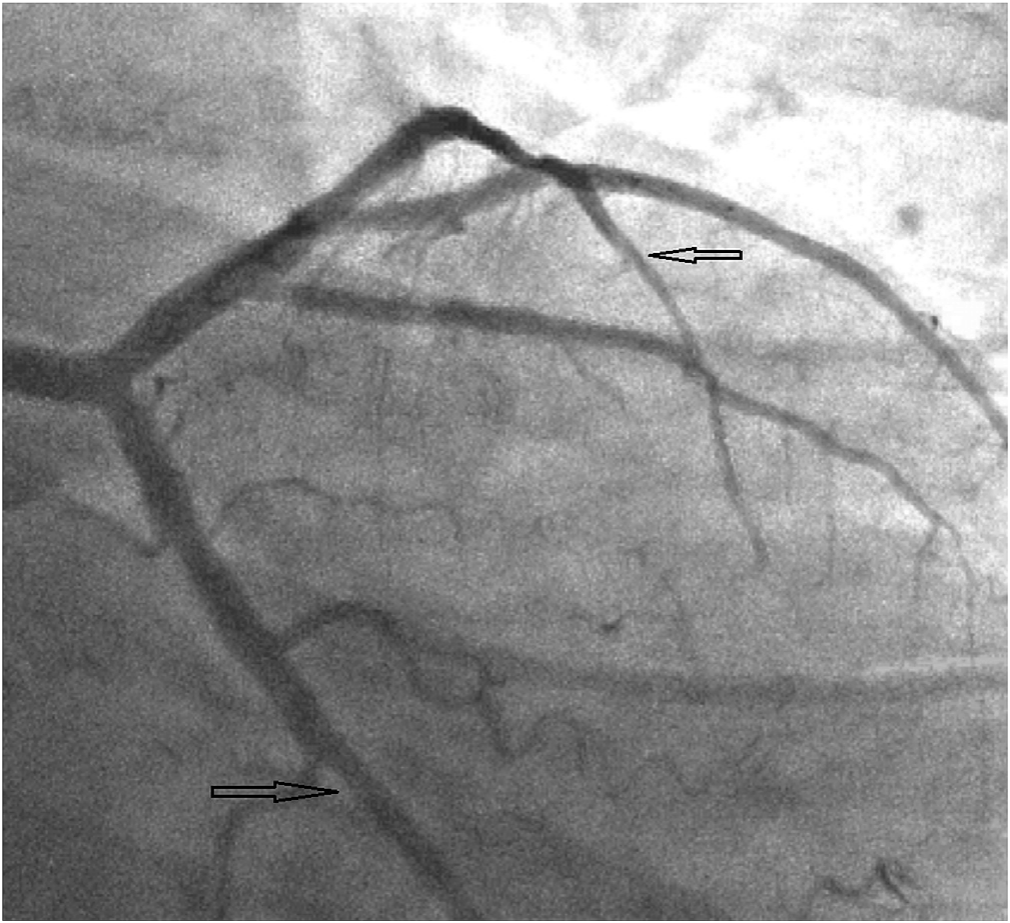
Residual changes of LAD and CA dissection.

According to the radionuclide perfusion scintigraphy results, it was decided to perform CAG, which revealed the Left main was without significant changes, in the distal segment of the LAD stenosis up to 40% was revealed, in the distal segment of CA - stenosis up to 30%, right coronary artery (RCA) was without significant changes (Fig. 4, Fig. 5).

Taking into account the results of these studies, it was decided to refrain from performing the operation regarding deforming osteoarthritis of both hip joints. These angiographic studies can be assessed as spontaneous recanalization of occluded segments with residual effects of eccentric stenosis in the area of previously detected occlusion.

## 3 Discussion

Considering the particularities of this clinical case, we assumed that myocardial infarction developed during cisplatin and capecitabine therapy for rectal adenocarcinoma.

One of the mechanisms of the SCAD during cisplatin and capecitabine (5-fluorouracil) consumption can be vasospasm. Chemotherapy based on the cisplatin usage, causes oxidative stress, reduces the activity of antioxidants and their concentration in plasma, that leads to the cardiotoxic effect development in the early stages of treatment [5,6].

The usage of cisplatin can cause the SCAD development, even when the other coronary risk factors are absent. One of the most important side effects of chemotherapy is cardiotoxicity. Completely, this applies to 5-fluorouracil, which causes a wide range of cardiovascular abnormalities, like arrhythmias or cardiopulmonary insufficiency, in particular, during capecitabine intake complications occur in 7,6% of cases [7].

Many investigations note the essential role of 5-fluorouracil in the development of vasospasm, that is a key risk factor of the SCAD [8,9]. Patients with existing cardiovascular pathology have a high risk of this disease [10]. Some cases of myocardial infarction, sudden death and cardiac arrest associated with the capecitabine intake have been described [11,12].

We've found publications of individual cases of SCAD associated with antineoplastic therapy. The Canadian Journal of Cardiology describes a case of SCAD, which led to acute myocardial infarction in a 24-year-old man after a course of cisplatin chemotherapy for testicular cancer. During coronary angiography, a double false lumen was identified in the right coronary artery (RCA), which was characterized as a chronic dissection of RCA [13]. A similar mechanism for the development of SCAD has been reported in the case of capecitabine intake. Particularly, a case of cervical cancer in a 39-year-old woman who suffered a myocardial infarction with ST-segment elevation during 5-fluorouracil treatment was reported. The angiography revealed the presence of extensive stratification of the coronary artery, which, according to the authors, was, at least partially, the result of spasm [14]. Another author describes a case of SCAD in a 69-year-old man with myocardial infarction who underwent surgery for colon adenocarcinoma and then received four courses of chemotherapy (oxaliplatin, capecitabine) [15].

## 4 Conclusion

In most cases, SCAD is diagnosed on the ground of autopsy data. After the introduction of modern imaging techniques into our practice, such as coronary angiography, intravascular ultrasound, optical coherence tomography, the diagnostics of SCAD improved.

The treatment strategy for SCAD includes conservative treatment, percutaneous coronary or surgical intervention. Some authors report that asymptomatic patients with non-occlusive dissection may be treated conservatively [14]. Conservative therapy includes aspirin, clopidogrel, beta-blockers in patients with a stable, asymptomatic form of limited dissection. It is preferable to avoid fibrinolytic drugs, since they can cause bleeding or progression of dissection.

The usage of nitroglycerin can control vasospasm of CA [[15], [16], [17], [18]]. Koller et al. used corticosteroids and cyclophosphamide in a multifocal inoperable case of SCAD and two months later, the angiographic signs of SCAD disappeared. The patient was observed for thirty months, no symptoms of SCAD were detected [19]. Cheung reported on the successful therapy with glycoprotein inhibitors with dissection resolution, which was observed after 20 hours on the control CAG [20].

According to the literature, it is known about the treatment of patients with SCAD who underwent balloon angioplasty without stent implantation [[21], [22], [23], [24], [25]]. However, coronary stenting is the preferred treatment for patients with single vascular SCAD, without involving the left main stem, and for patients with acute coronary syndrome or recurrent ischemia, due to the ability to restore the true lumen of the artery [18,21,22,[26], [27], [28], [29]]. PCI can be considered in cases when a lesion of large vessels is detected. The literature describes cases of stenting in mono- and multifocal dissection with good long-term results [[21], [22], [23], [24], [25], [26], [27], [28], [29]]. In such cases, the stent implantation should be performed extremely carefully and within the unaffected areas of the artery due to the possibility of dissemination in the distal and proximal directions. There are some cases when the stent implantation provoked the development of additional dissection and thrombosis [30].

In case of multivessel lesions of the coronary arteries or/and with involvement of the left main coronary artery it is desirable to perform coronary artery bypass grafting [31].

Thus, SCAD is a disease with an extremely complex etiololy, which does not have a special treatment guide. Management should be considered individually for each case. First of all, it is necessary to identify and eliminate the provoking factor of SCAD. Anticancer therapy is used in everyday practice quite often. However, most drugs are characterized by high level of cardiotoxicity. Many studies have been conducted and the majority of them note the important role of 5-fluorouracil in the development of vasospasm - a key risk factor for the SCAD.

## Provenance and peer review

Not commissioned, internally reviewed.

## Ethical approval

Written consent was taken from the patient.

## Sources of funding

Pirogov’s National Medical Surgical Center Moscow, Russian Federation.

## Author contribution

Somov Pavel: data collections, data analysis and writing.

Dmitriy Marchak: data collections and data analysis.

Arkhip Matusov: data analysis and writing.

Alexander Viller: data collections.

Archili Miminoshvili: writing.

Yuri Shevchenko: study design.

## Conflicts of interest

Non-Financial Conflicts of Interest:

## Research registry number

Not required.

## Guarantor

Somov Pavel.
