# Supplementary material for: Spontaneous coronary artery dissection during cisplatin and capecitabine therapy
Source: Ann Med Surg (Lond). 2019 Jul 11;45:1–5. doi: 10.1016/j.amsu.2019.07.018 (PMC6626886; doi:10.1016/j.amsu.2019.07.018)
Supplement: Multimedia component 1 [file mmc1.docx]

| **SCARE Checklist** | | | |
| --- | --- | --- | --- |
| **Topic** | **Item** | **Checklist item description** | **Page Number** |
| **Title** | **1** | Сase report of spontaneous coronary artery dissection during cisplatin and capecitabine therapy. | 1 |
| **Key Words** | **2** | Spontaneous coronary artery dissection, myocardial infarction, capecitabine, cisplatin. | 1 |
| **Abstract** | **3a** | SCAD is a rare cardiovascular disease that can cause acute myocardial infarction and sudden cardiac death. The mechanism of this pathology and the optimal treatment are not fully understood. | 1 |
|  | **3b** | Coronary angiography demonstrated a multivessel occlusion of coronary arteries. |  |
|  | **3c** | The main diagnoses - myocardial infarction, total hip arthroplasty, rectal adenocarcinoma. |  |
|  | **3d** | SCAD is a disease with an extremely complex etiololy. The authors discuss several factors that may lead to the spontaneous coronary artery dissection including chemotherapy-induced vasospasm. |  |
| **Introduction** | **4** | Spontaneous coronary artery dissection (SCAD) is a very rare cardiovascular disease that due to the stratification of the coronary artery wall with a false lumen formation can lead to acute myocardial infarction (AMI) or less often to a rhythm disturbance and sudden heart death. Federal State Medical Surgical Center. | 1 |
| **Patient Information** | **5a** | 55y.e. Female. East Slavic ethnic. BMI 29.38 А ( II ) Rh + | **2** |
|  | **5b** | In August 2014, the patient suddenly felt a chest pain for the first time in her life. Brought into Emergency room by ambulance. |  |
|  | **5c** | Cr rectum T3N1M0 after surgical treatment. Chemotherapy(November 2013.) Closure of the bladder with re-resection of the rectum (May 2014) |  |
|  | **5d** | No smoking. autonomic activity, but restriction of joint movement |  |
| **Clinical Findings** | **6** | Describe the relevant physical examination and other significant clinical findings (include clinical photographs where relevant and where consent has been given). | 2 |
| **Timeline** | **7** | Urgent echocardiography and coronary angiography 2014 year. radionuclide perfusion scintigraphy coronary angiography 2016 year | 2 |
| **Diagnostic Assessment** | **8a** | radionuclide perfusion scintigraphy coronary angiography | 2 |
|  | **8b** | no challenges |  |
|  | **8c** | myocardial infarction, Cr rectum T3N1M0, SCAD |  |
|  | **8d** | T3N1M0 |  |
| **Therapeutic Intervention** | **9a** | Anticoagulation medications | 3 |
|  | **9b** | toshiba angiography machine |  |
|  | **9c** | Radial approach. Local anesthesia. |  |
|  | **9d** | Somov Pavel 6 years experince |  |
|  | **9e** | multivessel coronary artery occlusion |  |
|  | **9f** | unchanged puncture site |  |
| **Follow-up and**  **Outcomes** | **10a** | no follow-up | 4 |
|  | **10b** | radionuclide perfusion scintigraphy in one year |  |
|  | **10c** | Tolerance is satisfactory |  |
|  | **10d** | No complications during hospitalization.5 |  |
| **Discussion** | **11a** | The mechanism of this pathology did not fully understood. | 6 |
|  | **11b** | Some cases of myocardial infarction, sudden death and cardiac arrest associated with the capecitabine intake have been described, many studies have been conducted and the majority of them note the important role of 5-fluorouracil in the development of vasospasm - a key risk factor for the SCAD. |  |
|  | **11c** | These angiographic studies can be assessed as spontaneous recanalization of occluded segments with residual effects of eccentric stenosis in the area of ​​previously detected occlusion |  |
|  | **11d** | Management of SCAD should be considered individually for each case. |  |
| **Patient Perspective** | **12** | Taking into account the results of these studies, it was decided to refrain from performing the operation regarding deforming osteoarthritis of both hip joints. A patient was discharged in satisfactory condition. | 7 |
| **Informed Consent** | **13** | Yes, consent is not obtained |  |
| **Additional Information** | **14** | All authors have no conflicts of interest to disclose.  The authors of this article did not have sponsorship. |  |
